# Supplementary material for: The Systems Biology Research Tool: evolvable open-source software
Source: BMC Syst Biol. 2008 Jun 29;2:55. doi: 10.1186/1752-0509-2-55 (PMC2446383; doi:10.1186/1752-0509-2-55)
Supplement: Additional file 1 — SBRT Archive. An archive of the current version of the Systems Biology Research Tool. [file 1752-0509-2-55-S1.zip › sbrt-1.4.0/doc/developers_guide/api/sbrt/shell/io/InputFile.html]

InputFile


|  |  |  |
| --- | --- | --- |
| **Overview** \| **Package** \| **Class** \| **Use** \| **Tree** \| **Deprecated** \| **Index** \| **Help** | |  |
| **PREV CLASS**   **NEXT CLASS** | **FRAMES**    **NO FRAMES**     **All Classes** |  |
| SUMMARY: NESTED \| FIELD \| CONSTR \| METHOD | DETAIL: FIELD \| CONSTR \| METHOD |  |


---


## sbrt.shell.io Interface InputFile<E>

**Type Parameters:**: `E` - the type returned by iterators over this type of input file.

**All Superinterfaces:**: java.lang.Iterable<E>, SbrtFile

**All Known Subinterfaces:**: MapInputFile<K,V>, MapTextInputFile<K,V>

**All Known Implementing Classes:**: AbstractMapInputFile, ComiFile, FluxCapInputFile, FluxIntervalInputFile, ProcessInputFile, RomiFile, RxnNamExprSetInputFile, SerializedInputFile, SerializedMapInputFile, TextInputFile

---

``` public interface InputFile<E> extends SbrtFile, java.lang.Iterable<E> ```

This interface is used to represent input files.
Input files are files that serve as input sources.
The contents of an input file are accessed via an
`java.util.Iterator` over the file.

**Author:**
:   This interface was written and documented by
    Jeremiah Wright while in the Wagner lab.

---

**Method Summary**

**Methods inherited from interface sbrt.shell.io.SbrtFile**

`close, getCanonicalName, getOriginalName, isClosed`

**Methods inherited from interface java.lang.Iterable**

`iterator`

---


|  |  |  |
| --- | --- | --- |
| **Overview** \| **Package** \| **Class** \| **Use** \| **Tree** \| **Deprecated** \| **Index** \| **Help** | |  |
| **PREV CLASS**   **NEXT CLASS** | **FRAMES**    **NO FRAMES**     **All Classes** |  |
| SUMMARY: NESTED \| FIELD \| CONSTR \| METHOD | DETAIL: FIELD \| CONSTR \| METHOD |  |


---
